# How society publishers can accelerate their transition to open access and align with Plan S

**DOI:** 10.1002/leap.1272

**Published:** 2020-01-13

**Authors:** Alicia Wise, Lorraine Estelle

**Affiliations:** ^1^ Information Power Ltd Winchester UK

**Keywords:** open access, Plan S, learned society publisher

## Abstract

Wellcome, UK Research and Innovation, and the Association of Learned and Professional Society Publishers commissioned Information Power Ltd. to undertake a project to support society publishers to accelerate their transition to open access (OA) in alignment with Plan S and the wider move to accelerate immediate OA. This project is part of a range of activities that cOAlition S partners are taking forward to support the implementation of Plan S principles. The objective of this project was to explore with learned societies a range of potential strategies and business models through which they could adapt and thrive under Plan S. We consulted with society publishers through interviews, surveys, and workshops about the 27 business models and strategies identified during the project. We also surveyed library consortia about their willingness to support society publishers to make the transition to OA. Our key finding is that transformative agreements emerge as the most promising model because they offer a predictable, steady funding stream. We also facilitated pilot transformative agreement negotiations between several society publishers and library consortia. These pilots and a workshop of consortium representatives and society publishers informed the development of an OA transformative agreement toolkit. Our conclusion is that society publishers should consider all the business models this project has developed and should not automatically equate OA with article publication charges.

**Key points**

- Experiences of open access (OA) publishing did not differ substantially between STEM and HSS learned societies that responded to the project survey.
- There is a tendency to conflate OA publishing with the article publication charge (APC) model and not consider other business models.
- Of the 27 different approaches to OA publishing, only 3 relied on APCs.
- Society publishers are most positive about transformative agreement models but are concerned about the ability to access and negotiate directly with library consortia.
- Learned societies need to communicate challenges and opportunities within their communities and with their stakeholders so that solutions can be identified and allies developed.

## INTRODUCTION

Wellcome, UKRI, and the Association of Learned and Professional Society Publishers (ALPSP) commissioned Information Power Ltd. to help learned society publishers thrive as they align with Plan S and the wider transition to immediate open access (OA). This was in response to concerns expressed in response to Plan S, Wellcome's future OA policy (as the first funder policy to incorporate the Plan S principles) and other related developments by learned society publishers reliant on the hybrid OA publishing model.

COAlition S, the consortium of research funders, launched Plan S in September 2018 and subsequently revised its requirements in early 2019. One important feature of the current Plan S guidance is that funders will not fund Article Publication Charges (APCs) for hybrid OA journals unless the journal is part of a transformative agreement. Transformative arrangements are described on the cOAlition S website (http://www.coalition-s.org) as strategies to transition to full OA and are envisaged to achieve full OA by December 2024, the date by which funding for hybrid journals within these arrangements will cease. After this date, hybrid journals may continue, but there will be no funding to pay APCs, and researchers publishing in such journals (as well as in subscription journals) will be required to deposit their accepted (post‐peer review) manuscript in a suitable repository at the time of publication, without embargo, under a CC‐BY licence. There are publisher concerns that the availability of such articles will undermine the viability of subscription journals. Information Power was therefore tasked with identifying methods that could be used by society publishers to comply with Plan S requirements.

## METHODS

We first undertook interviews with society publishers to explore a range of strategies and business models that would help them comply with the requirements of cOAlition S. We then published a discussion document (Wise & Estelle, 2019a, 2019b) outlining 27 strategies and business models all compliant with Plan S. The survey asked society publishers to rank their interest in these models and sought their more detailed feedback through free‐text fields. The survey was open from 25 March 2019 until the end of June 2019. The survey questions and anonymized response data are available on the Wellcome Trust Figshare platform. As with all surveys, there is the risk of a self‐selection bias. However, this was mitigated by links to the survey being distributed via the ALPSP and via social media, and we also asked that larger publishers providing services to learned societies support us by distributing the questionnaire.

In addition to the online survey of society publishers, the first project workshop was hosted by the Wellcome Trust and took place on 26 April 2019. The 21 participants included representatives of 12 learned society publishers, other members of the project steering group, and Information Power consultants. The learned society publishers were evenly split between STEM and humanities and social science (HSS) subjects, but in HSS, there were predominantly social science publishers. A full account of this workshop is included in the final project report (Wise & Estelle, 2019c).

As part of this project, we also surveyed library consortia about their willingness to help society publishers to make the transition to OA. The survey was open from 21 February 2019 until mid‐April 2019. A total of 26 library consortia participated, and the results of this part of the project and the subsequent workshop of consortium representatives and publishers have been published (Wise & Estelle, 2019d). Our conclusions were that, while library consortia have limited resources, they are positive about engaging with learned society publishers and helping them transition to new models. Library consortia would like to see OA accelerated and look forward to new, transparent business models for journals.

We then held a second workshop in London inviting an international array of library consortia leaders and learned society publishers. This workshop agreed on principles for transformative agreements. Following this workshop, several consortia and publishers agreed to work with us to pilot agreements based on the principles. Pilot participants were Brill, the Council of Australian University Libraries, the European Respiratory Society, IWA Publishing, Jisc, the Max Planck Digital Library, the Microbiology Society, Portland Press, and the Association of Universities in the Netherlands.

Facilitating the discussions between learned society publishers and library consortia made us realize that there was a need for a toolkit. This toolkit should be freely available and fully adaptable to save stakeholders from duplicating efforts in identifying routes and solutions. The toolkit, created as an output of this project, contains a ‘tips and tricks’ guide to negotiating a Transformative Agreement; an Excel spreadsheet for collecting the historic data needed to price a Transformative Agreement; an Overview Document, setting out both the spirit of the agreement and the key practical issues; and, finally, a Model Licence for the Transformative Agreement (Wise & Estelle, 2019e).

## SURVEY RESPONDENTS

We received 105 responses to the survey, primarily from the UK (64) and USA (23), but we received welcome responses from societies based in China (4), the Netherlands (3), Sweden (2), Belgium (1), France (1), Germany (1), and Switzerland (1). One society was international, the focus of operations changing as each new president is elected. One respondent was an umbrella organization based in the Netherlands with 30 national member societies.

Of our respondents, 72% were societies who publish via larger publishing partners, and 28% were independent society publishers. This emerged as a very important distinction both when analysing the survey results and later in issues and opportunities that arose during workshops. We expected that one really important distinction in our results would relate to whether the society respondent published in STEM subjects or HSS subjects. There were indeed some differences but not nearly as many as we had foreseen. A total of 63 respondents identified as STEM societies, 30 as HSS societies, 7 as both, 3 as ‘other’, and 2 skipped this question.

### Key differences between STEM and HSS societies

**Figure 1:**
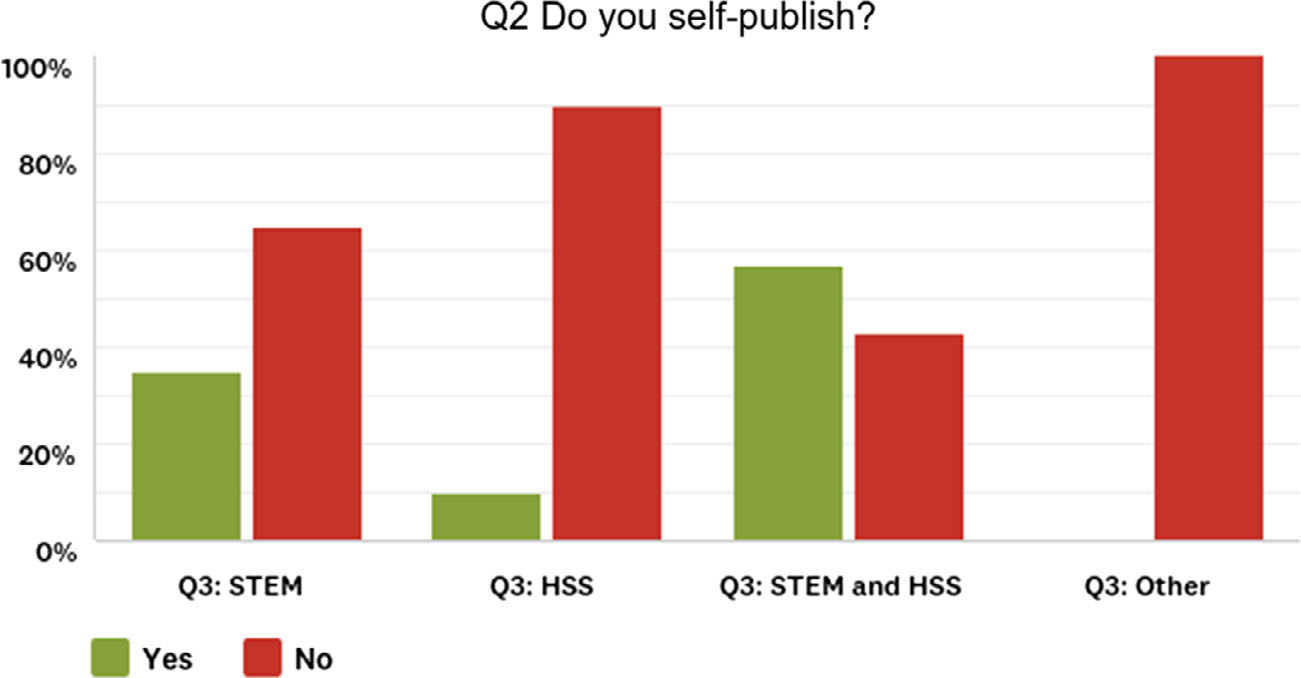
Responses to question 2 of the survey, which asked if publishers self‐publish (*n* = 103). Q3 in the legend indicates the question we used as a filter to distinguish the publisher types: STEM, HSS, STEM and HSS, and other.

We first analysed the online survey of society publishers to check if there were differences between respondents who identified as STEM or HSS societies. One difference that emerged is that a higher number of the independent society publishers were in STEM disciplines, and there were virtually no independently publishing HSS respondents as show in Fig. 1.

**Figure 2:**
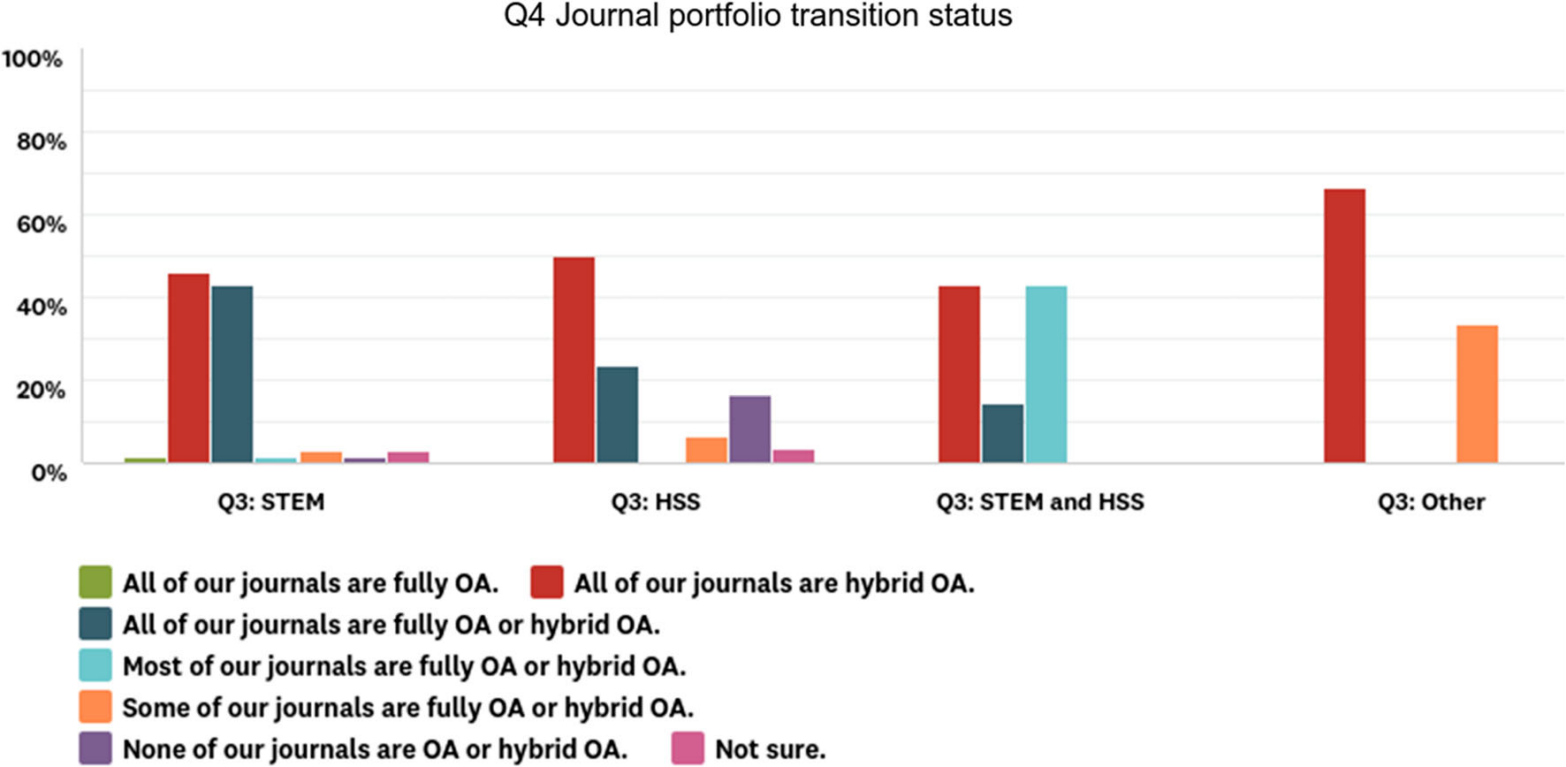
Responses to question 4 of the survey, which asked about the transition status of the respondent's journals (*n* = 103). Q3 in the legend indicates the question we used as a filter to distinguish the publisher types: STEM, HSS, STEM and HSS, and other.

We expected a large degree of difference in experience of OA publishing between STEM and HSS learned societies and were surprised to discover that this was not the case. Only one STEM publisher and five HSS publishers reported that none of their titles were fully or hybrid OA. There was a modest difference between the disciplines with regard to having experience with fully OA titles versus hybrid OA titles. Around 50% of both STEM and HSS respondents reported that all of their titles are hybrid OA. An additional 45% of STEM publishers reported that all of their titles were either hybrid or fully OA, while only an additional 23% of HSS publishers reported this (see Fig. 2).

We probed our data carefully for differences between HSS and STEM publishers and found relatively little except for the large number of HSS society publishers who have publishing partners. However, throughout this project, we have heard from every HSS society we have engaged with that HSS is entirely different from STEM and that they would appreciate our emphasizing this in our conclusions and to funders. We were left wondering if this just reflects the tendency to treat the APC funding model and OA generally as if they were the same thing – and the smaller proportion of HSS authors with access to funding for APCs – which we detected in the course of our study. There appears to be no difference in the way the OA business models and transition strategies we have identified can be applied to HSS and STEM publishers.

### What we learned from society publishers through our survey

While the APC is the best‐known business model for OA journals, there has been a tendency to treat APC and OA as if they were the same thing. It is a problematic business model upon which to base a wholesale transition of hybrid titles to OA because not all authors have access to funding to pay for APCs or would be willing to do so even if they did. This is a challenge that impacts all publishers seeking to transition to hybrid titles, whether they publish in HSS or in STEM fields.

If publishers, including society publishers, are going to make a sustainable transition to OA publishing – as many in the course of our project stated they wish to do – then they cannot simply rely on the APC business model or indeed on any other transactional payments by authors. They need to transform other existing revenue streams to support OA publishing.

We are convinced that this is possible with attention and focus and that outsourcing to larger publishing partners is *not* the only sustainable strategy available to societies.

From the 27 different approaches and business models we identified during this project, only three relied on transactional payments by authors. All these 27 models support full, immediate OA and are Plan S‐compatible. They can be used alone or in combination. For ease, we have clustered them together into seven categories: transformative models, cooperative infrastructure and funding models, immediate sharing with open licence models, article transaction models, open publishing platforms, other revenue models, and cost reduction. Table 1 summarizes the approaches and models and provides examples.

**Table 1:** Approaches and business models currently in use to provide open access publications with examples.

Transformative models	Cooperative infrastructure + funding models	Immediate sharing with CC‐BY licence	Article transaction models	Open publishing platforms	Other revenue models	Cost reduction
			Examples			
California Digital Library pilot transformative agreement	Hrčak, Croatia	Author self‐archiving	APC‐funded OA	Wellcome Open Research	Advertising	Close or combine journals
Knowledge Unlatched journal flipping programme	Kotilava		Institutional prepay models with partially discounted APCs	Gates Open Research	Crowdfunding	Cooperative infrastructure
Libraria	Open Library for the Humanities		Submission payments	F1000	Subsidies	Increase article numbers
Publish‐and‐read agreements	Project MUSE			Emerald Open Research	Freemium	Online‐only publishing
Read‐and‐publish agreements					Syndication	Outsourcing
SCOAP^3^						Partner
Subscribe to open						

### Transformative models

These approaches repurpose existing institutional spend with publishers in order to open content. They are promising transition models because libraries and library consortia provide the lion's share of funding in the current publishing landscape. If this revenue stream is transformed to support OA, then journals can also transform to be fully OA. Institutional and consortia agreements are easier to administer than hundreds or thousands of author payments and provide an attractive predictable flow of revenue. They are also helpful models for publishers to use to align with Plan S because hybrid journals within transformative agreements have more time in which to transition to full and immediate OA.

At least seven types of transformative agreement operate in the market today:

#### California Digital Library pilot transformative agreement

This model engages authors and libraries. The library or consortium contributes money in the form of a direct payment to the publisher in order to lower or subsidize transactional publishing payments by authors who can afford to contribute something towards the cost. This approach is designed to reflect the fact that researchers in the USA can use their research grants to pay for publication costs if they choose to do so but are usually under no obligation or mandate to do so.

#### Knowledge Unlatched journal flipping programme

This is sometimes termed a choreographed transition model. In this case, Knowledge Unlatched (http://www.knowledgeunlatched.org/) acts as the choreographer. Librarians pledge continued funding for titles that publishers then pivot to publish OA. No APCs are charged, and all funding comes from participating libraries.

#### Libraria

This approach (see http://libraria.cc/), which is being piloted in anthropology, archaeology, and neighbouring fields, involves pooled money from funders and libraries being used to fund OA publishing. The journals are long established and will transition fully to OA when this funding is secured.

#### Publish‐and‐read agreements

A consortium pays a pre‐agreed amount for papers published by affiliated authors, and everyone in the library/consortium receives access to the subscription content for no extra cost. The agreement between Wiley and Projekt DEAL in Germany is one example (see http://www.projekt-deal.de/wiley-contract/).

This model shifts the cost basis of publishing to align with the number of articles involved. For this reason, it may be challenging for consortia in research‐intensive countries and/or their members in research‐intensive institutions. The difficulty is that a consortium will have to agree with its members on a fair method of redistributing the total cost because the most research‐intensive institutions are likely to pay significantly more than they do under the subscription model, and less research‐intensive institutions are likely to pay significantly less. There will be winners and losers to manage, and so, a more gradual approach to rebalancing or a broader basis on which to calculate and apportion costs could be helpful.

#### Read‐and‐publish agreements

The amount of money currently paid to the publisher (for subscriptions and sometimes also for APCs where there has been additional funding for OA publishing) is guaranteed, and in exchange, authors can publish OA without paying an APC. In some instances – for example, where a country publishes many articles with a publisher, or an increasing number of articles is being submitted to the publisher from authors in that country – additional money may sometimes be made available by libraries or consortia. Consortia and their members are price sensitive, however, and will sometimes cap the total number of articles for which they will pay in order to control costs.

Examples include consortia arrangements in the Netherlands, Sweden, the UK, and at MIT, with publishers such as IOPP, OUP, the Royal Society of Chemistry, and Springer Nature via Springer Compact (see http://www.springer.com/gp/open-access/springer-open-choice/springer-compact).

##### SCOAP^3^

This is also what might be termed a choreographed transition model with CERN serving as the choreographer with diverse dancers (or journals) to align. Participants include libraries, consortia, governments, publishers, societies, and researchers (see https://scoap3.org/).

The basic idea is that current library spend is directed to CERN rather than the publisher. CERN calculates the proportion of high‐energy physics articles in participating titles that come from each country and assesses whether current library spend covers that country's participation or needs to be topped up in some way. If necessary, it liaises with national funders and policymakers about top‐up funding. CERN then uses the funding pool to pay the APCs of all authors in participating titles. Publishers flip these titles to be fully OA rather than published on a subscription or hybrid basis.

The complexity of this approach means that it has been used on a modest number of journals, but it has made a real impact as all the journals are concentrated in high‐energy physics. Stakeholders make this work and collaborate to resolve issues as they arise. One challenge is that, to ensure clarity of costs for all funding participants, article numbers are sometimes capped, which can cause problems for publishers whose titles are growing organically as they increase their appeal to researchers.

### Subscribe to open

This approach has been developed by the publishing team of the non‐profit publisher Annual Reviews (http://www.annualreviews.org/). It is designed to motivate collective action by libraries, which are asked to continue to subscribe even though the content will be published OA. A 5% discount off the regular subscription price is offered to existing customers. If all current customers continue to subscribe, then that year's content is made available on OA, as are all the backfiles. None of this content is opened if the number of subscribers declines, which discourages free riding. The subscriber base will be expanded to offset attrition, which is currently 1–2% per year. There is no library lock‐in as this offer is repeated each year, and customers again decide whether they wish to continue subscribing. If participation levels are insufficient to open the content in any given year, the 5% discount is still extended to customers, but for that year, the journal will not be Plan S‐compliant. Any institutions that do not renew and that later return do so at the list subscription price and do not receive the 5% discount.

Annual Reviews piloted this model with one title and received a 25% increase in citations and a 300% increase in downloads. These downloads were not only from the users of the 2,000 subscribing institutions but also from a further c. 7,000 institutions whom Annual Reviews can now approach with data about why they might wish to subscribe and support the journal. In 2020, Annual Reviews will extend this model to five journals or 10% of its portfolio.

This model – uniquely among the Transformative Agreement models – positions the publisher as the choreographer of change. It leverages the conventional subscription process and existing library budgets, avoids the need to invest in transactional payment infrastructure, minimizes customer disruption by using routine library accounts‐payable processes, and avoids the prohibition some libraries face in paying for things that would otherwise be free.

### Society publisher views of transformative models

**Figure 3:**
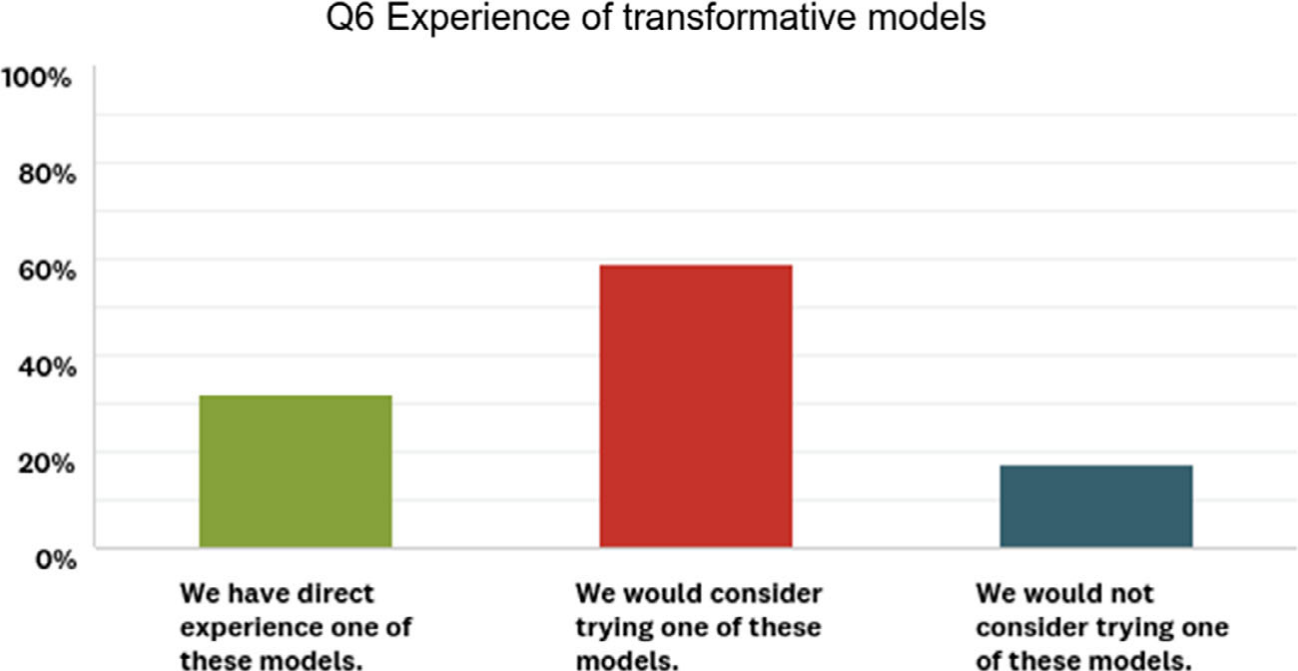
Responses to question 6 of the survey, which asked participants about their experience of transformative models (*n* = 102).

Transformative agreements were of interest to many society publishers who participated in our project, although only 30% of them had experience of such models (see Fig. 3). This is because this transition approach does not depend on authors having access to APC funds and because it produces a steady and predictable revenue stream in just the same way that traditional subscriptions have done.

A major practical concern for society publishers was the ability to access and negotiate with library consortia, and so, the results from our survey of consortia that showed support for working in this way with smaller publishers should provide some welcome reassurance. Several additional practical challenges with regard to transformative agreements were identified during our survey.

- Society publishers need opportunities to learn about transformative agreements ***very quickly*** and to refine/reject their pitches quickly to align with Plan S deadlines. Pilots in 2019 would have been desirable, to allow an entire renewal cycle to be run through in 2020, before Plan S implementation begins in earnest.
- Society publishers desire clarity about what a Plan S‐compliant transformative agreement looks like and what data are needed in order to enter a constructive negotiation with consortia or libraries.
- Society publishers want confidence that an approach to transformative OA agreements would resonate with libraries and that they would gain traction in the market.
- Society publishers are curious about rebalancing the approaches that consortia might take and how any changes will be phased in as this might inform their own pricing models and approach to transformative agreements.

### Cooperative infrastructure + funding models

These are close, strategic partnerships between libraries and publishers to jointly fund and provide open content and its supporting infrastructure. These models are deployed successfully in HSS publishing.

There are several examples of close cooperation between libraries and publishers to agree on both shared infrastructure and shared approaches to funding publication costs. Currently, it appears that this model is particularly useful in countries with a strong strategic focus on culture and language and in HSS subject areas. Example initiatives are: Hrčak (https://hrcak.srce.hr/), Kotilava (http://kotilava.fi/19-elokuu-2016-1247/kotilava-%E2%80%93-finnish-academic-journals-towards-immediate-open-access), Open Library for the Humanities (http://www.openlibhums.org/), and Project MUSE (https://muse.jhu.edu/).

#### Society publishers' view of cooperative infrastructure + funding models

There was less enthusiasm for this approach from societies within the UK and USA, and a review of full‐text comments suggests that concerns fell into two categories. The first might be described as *agency* concerns. Fourteen respondents said they would need advice from their publishing partner in order to evaluate this approach and what it could mean; if it seemed as if it might be relevant, they would then need their publishing partner or some other organization to develop, lead, and organize such a partnership. The second set of concerns related to *scalability and sustainability*, expressed by 16 respondents. These comments highlighted the importance to many publishers of having OA business models that would work globally for authors and readers and that would be sustainable over time, with predictable annual revenue streams. They indicated that other business models were more likely to deliver against these criteria during a full transition to OA.

Of the 104 respondents that answered this question, less than 17% were extremely interested or very interested in this approach. Participants had indicated in which country their head office was located, and a review of the individual responses to the survey showed that 8 of the 15 respondents from outside the UK and USA were extremely or very interested in exploring this approach further. One respondent from China believed the approach would work well there because of strong local‐language publishing, which is often already centrally funded.

### Immediate sharing via self‐archiving under a CC‐BY licence

It is possible to continue to operate journals fully funded by the subscription model and comply with Plan S by permitting authors to immediately self‐archive their accepted manuscripts or final articles under a CC‐BY licence. This green OA approach is dependent on either final published journal articles or author‐accepted manuscripts being shared with a CC‐BY licence at the time of publication.

#### Author self‐archiving

The subscription model entirely funds this approach to OA, and so, an important consideration is what will happen to the subscription payments if all, or even a majority, of the journal's content is available in this way. Some publishers view this as challenging because a small minority of titles have a usage half‐life of less than 12 months (Davis, 2013; Publishers Association, n.d.), and usage data are important to librarians when making purchasing decisions.

A number of publishers – including society publishers – have, however, deployed 0‐month embargos without complaining of lost revenue or other negative impacts (see the Google doc created in November 2018 – Anon, 2018). One publisher shared with us in confidence that they had trialled a 4‐week embargo period and attributed lost subscriptions to this trial, but this was the only negative anecdote. A possible approach in the first instance could be to use a 0‐month embargo period and CC‐BY licences only for authors funded by the funding bodies participating in cOAlition S. If subscription revenue remains stable, 0‐month embargos could be rolled out more broadly.

#### Society publisher views on immediate self‐archiving with CC‐BY licence

This approach was surprisingly popular with respondents, with nearly half extremely or very interested.

The full‐text comments added to the survey provided helpful context and demonstrated that publishers may be viewing this more as a potential short‐term response to Plan S. Many respondents, including those who were extremely or very interested, questioned whether it would be a sustainable model. It would depend on whether or not libraries continued to subscribe when a large proportion of a journal's content was openly available, and that is unknown.

Our survey results suggest slightly more anxiety about the sustainability of this approach from learned societies with larger publishing partners. Perhaps ‘big deal’ packages are more susceptible to cancellation in such an environment. There was also slightly more anxiety about the sustainability of this approach from learned society publishers based in the USA, but it was unclear why there might be a geographic variation.

### Article transaction models

Author payments such as APCs and submission fees can work perfectly well to underpin an OA transition strategy in titles where the large majority of authors are well funded and support such payments. These models might work for a society publisher with a steady flow of articles and the infrastructure to administer many small transactions.

#### APC‐funded OA

Content is published OA because publishing costs are covered by APCs typically made by a researcher, their funder, or their institution. This is a proven model and works best in well‐funded discipline areas with strong researcher support for OA publishing. It is a way of making the price of publication more transparent to researchers but can be expensive for both libraries and publishers to administer because of the number of transactions it involves.

There are ethical issues to manage with any pay‐to‐publish model and real and perceived risks of lower standards or vanity publishing by unscrupulous organizations claiming to be proper publishers. To counter this, the Directory of Open Access Journals (DOAJ) has established helpful standards and best practice for OA journals and publishing. Qualification for indexing in DOAJ is often a prerequisite for membership in organizations such as the Open Access Scholarly Publishers Association.

#### Institutional prepay models with partially discounted APCs

Libraries or consortia pay an upfront fee to the publisher in exchange for a discounted APC for themselves or for affiliated authors. This model can also operate at consortia level. When the discount reaches 100% and authors are no longer paying APCs at all, then there is not really a difference between this model for fully OA journals and a transformative agreement for formerly hybrid OA journals.

The OA articles published under such a prepay model are often deposited in an institution's repository. Examples include *Hindawi Open Access Membership* (https://about.hindawi.com/institutions/), *BioMed Central* (http://www.biomedcentral.com/about/institutional-support/membership), and *SpringerOpen Membership* (http://www.springeropen.com/about/institutional-support/membership) and the *Royal Society Open Access Membership Programme* (https://royalsociety.org/journals/librarians/subscribe/open-access-membership/).

#### Submission payments

These payments can be used in combination with another model to spread the cost burden between authors who submit articles that are rejected and those that are accepted. It appears to work for high‐quality and highly sought‐after titles with high rejection levels, and in some subject areas (e.g. economics), it has long been a normal practice.

This model appears to be under renewed consideration for a broader range of subjects, including STEM fields. A real concern that might inhibit any move to deploy this model more widely is that, unless all publishers were to switch to it at the same time, it would probably drive submissions to competitor titles.

Operating this model would perhaps be the easiest and most lucrative for large publishers with large ecosystems of journals. These publishers would be able to offer authors a high likelihood of being published somewhere in exchange for one submission payment or else would be able to collect multiple submission payments from each author.

#### Society publisher views on article transaction models

Our sense throughout the project has been that many participants confused OA publishing with the APC model, which is only one of many options for funding OA, and that they were also concerned about the uneven distributing of funding for APCs. Overall, 41% of our respondents were extremely or very interested in this model, but HSS society publishers were far more anxious, with only 18% of respondents saying that they felt extremely or very interested. Geography magnified this still further. Of our STEM respondents from the USA, 50% were extremely or very interested in this model, but not a single HSS respondent from the USA felt the same way (although this may reflect the small sample size, with only two US‐based HSS society publishers participating). Of our STEM respondents in the rest of the world (ROW), 75% were extremely or very interested, but again, not one HSS respondent from these geographies felt the same (and again, this may reflect the small sample size with only three ROW‐based HSS society publishers participating). Rather interestingly, UK respondents were slightly more sceptical about this approach, with only 37% feeling extremely or very interested; of this group, 50% of the STEM respondents and 19% of the HSS respondents were extremely or very interested. This probably reflects the outcomes from the Finch Review process (Finch, 2012), and the relative availability of APC funding for authors with grants from the (UK) Arts and Humanities Research Council and the Economic and Social Research Council.

Publishers who were interested in APC payments generally were also interested in institutional prepay schemes, but we detected little enthusiasm from institutions for these schemes. This is probably because the number of articles published with small‐ and medium‐sized publishers is likely to vary a good deal, and so, agreeing on precisely how many articles to pay for in advance is challenging.

A very small number of publishers expressed real interest in submission fees, with one respondent just poised to launch them for one of its journals. The anxieties expressed by other respondents were around the uneven availability of funding coupled with very serious concerns that it was unfair, or at least impractical, to expect authors to pay when they had a high chance of having their article rejected.

### Open publishing platforms

For the purpose of Plan S, OA platforms are publishing platforms for the original publication of research output (such as Wellcome Open Research, https://wellcomeopenresearch.org/ or Gates Open Research, https://gatesopenresearch.org/) and not platforms that aggregate grey literature or re‐publish content that has already been published elsewhere. Pioneered by F1000Researcg (https://f1000research.com/about) and first adopted by funders, this model is now being embraced by publishers. *Emerald Open Research* (https://emeraldopenresearch.com/) is one example.

In this approach, authors publish their articles, which are then openly peer reviewed. Societies adopting this model could, for example, provide peer review and/or curation services. Articles that are judged to be important and impactful can be specially curated and showcased. Funding for these services could be obtained through any of the OA business models we have identified. APCs are currently the most common.

In the F1000 model, post‐publication invited open peer review, and data services are provided by F1000 for a per‐article fee. Then, learned society publishers can create services such as overlay journals and charge for these services.

#### Society publisher views on open publishing platforms

**Figure 4:**
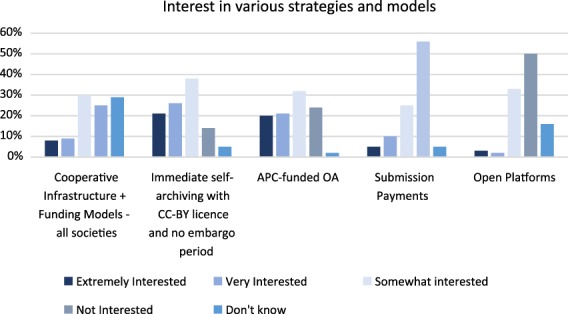
A summary of the responses to questions about interest in the various models (*n* = 102).

This model is creative, innovative, and intelligent but perhaps too little known to be popular. Over 63% of the 104 participants who responded to the survey question about interest in open platforms said that they were not interested or were only somewhat interested (see Fig. 4). It is certainly one model we believe is worth watching actively and experimenting with if at all possible.

### Other revenue models

There is a wide array of other business models that can work for individual publishers or titles. Examples include advertising; crowdfunding; bequests, donations, endowments, and subsidies; freemium; and syndication. Only one model seemed little known, and this is syndication.

The syndication business model is used in other creative industries, for example, in film and TV where one company produces the content and one or more other companies broadcast or stream it. Unique or valuable published content, for example, editorial front matter, could potentially be licensed to an array of publications and platforms rather than exclusively published in a journal. The licence might be granted in exchange for a fee or services and could be exclusive or non‐exclusive. One current example of this from the scholarly communication landscape is the licence publishers grant to indexing services in exchange for being indexed. The possible future extension of this model given the emergence of research ecosystems is a theme developed in Scholarly Kitchen blogposts by Roger Schonfeld (2019).

#### Society publisher views of other revenue models

**Figure 5:**
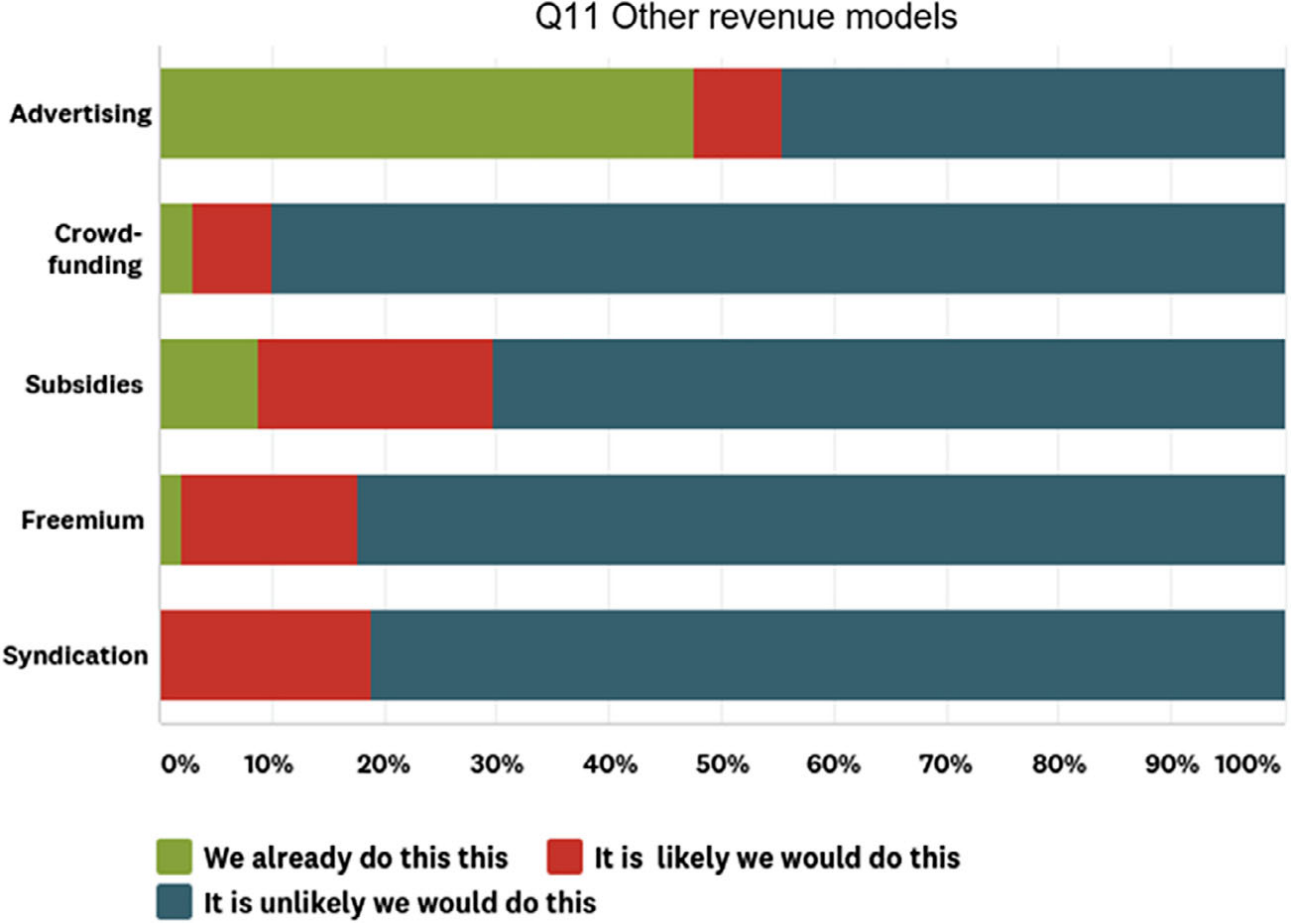
Responses to question 11 of the survey, in which participants told us if they already use other revenue models or are likely or unlikely to do so (*n* = 102).

Our respondents were largely familiar with these models, and – with the exception of syndication – these models have been widely tried and tested. They are viewed, with the exception of advertising, as a modest form of additional revenue rather than as realistic alternatives to the predictable and sustainable subscription revenue on which society publishers currently rely. There was little variation in response between STEM and HSS publishers, between independent publishers and those with publishing partners, or between publishers in different geographies (see Fig. 5).

### Cost reduction

Efficiency gains will help with a transition to OA, and there are some well‐established ‘tricks of the trade’ that remain viable, whether that is changing journals, collaborating, or outsourcing.

#### Close or combine journals

This approach is a potential way to reduce costs or to concentrate the proportion of authors able and willing to pay APCs into a single title. It can work at different levels, including across a single publisher's portfolio or across a consortium of publisher portfolios.

#### Cooperative infrastructure

There is currently a really vibrant landscape of cooperative infrastructure development and plenty of funding for developing open‐source software. Services are also beginning to emerge to help organizations without in‐house technological expertise implement and use these tools. A few examples include the following.

The Collaborative Knowledge Foundation (CoKo, https://coko.foundation/) is a not‐for‐profit cooperative development deploying open‐source infrastructure to support innovation in scholarly communications. It provides tools not only for journal publishing but also for books and micropublications. Active participants include OA‐only publishers *eLife* (https://coko.foundation/coko-and-elife-partnership/) and Hindawi (https://coko.foundation/coko-and-hindawi-partnership/).

These same organizations, and others including Digirati, are working together on Libero (https://libero.pub/), an innovative open‐source publishing environment to develop entirely new features, for example, tools to test for and demonstrate whether research is reproducible.

The Public Knowledge Project (PKP, https://pkp.sfu.ca/) has developed *Open Journal Systems* (OJS) with funding from a *wide array of organizations*. This open‐source publishing software is made freely available to journals worldwide for the purpose of making OA publishing a viable option for more journals and for more libraries and scholars who wish to self‐publish.

#### Increase article numbers

A rational approach to a fully OA world where money is available for authors to pay to publish is to increase your article market share. However, this can be a deeply unpopular transition strategy with funders and librarians when it is perceived to be done for financial gain rather than the benefit of researchers. Funders and libraries wish to transition to OA in a way that manages and reduces systemic costs (while expanding the content that is available and maintaining or improving quality), and they wish to encourage more competition in publishing. They do not wish to drive an arms race between publishers to see which can increase their market share of quality articles and price or (worse still) increase their market share by lowering quality standards.

#### Online‐only publishing

To save costs, learned society publishers may need or wish to move fully online. This can cause some challenges that need to be thought through carefully so that any lost revenue can be offset. For example, if print copies are a benefit offered to society members, membership fee revenue may be at risk if print copies cease to exist. In certain subjects, most notably in medicine, learned society publishers have significant advertising revenue tied to the print copies distributed to their members. In other cases, the benefits of moving to online only are likely to outweigh the disadvantages.

There can be opportunities too. One society publisher reported that moving online led to a modest increase in digital subscription sales because some fraudulent print subscriptions were cut off.

#### Outsourcing

Where societies have a publishing partner, they can benefit from existing expertise, infrastructure, and intelligence and might also be, to some extent, buffered by multi‐year contracts. The important thing is for learned society publishers to reflect on how they can best structure and drive these partnerships to enhance their society's mission and strategy. They are in the driving seat when procuring these services and can structure them to help drive change and innovation. There is a broad spectrum of publishing partners to consider, including the following:

***Other independent society publishers*** – The ALPSP learned journal collection, launched in 2008, was an early example in this space, and it is interesting to see the formation of new groups since the publication of Plan S. Examples include the Society Publishers Coalition (http://www.socpc.org/) and Transitioning Societies to Open Access (https://tspoa.org/).
***Library presses** –* If a learned society publisher is to maintain editorial independence, to really push the creative boundaries in online publishing, and to remain tied to the academic community in their publishing activities, then these new partners may be for them. Some offer publishing services, for example, the University of Michigan Press.
***Open‐access only publishers** – These organizations have a wealth of OA experience, services, and tools, so partnering with them can be a great way to accelerate. Larger publishers do this too, for example, Sage and Wiley with Hindawi*.
***University presses** –* These organizations come in all shapes and sizes, and many provide publishing services to learned society publishers. The largest have more experience, great scale, and experience in transitioning journals to OA, so they can offer a safety net and stability over multiple years. In return for a flat fee, profit share, or revenue share, learned society publishers can outsource some or all of their publishing and remain aligned with the academic community.
***Mixed‐model commercial publishers*** – With more experience, huge scale, and experience transitioning journals to OA, these partners can offer learned society publishers a safety net and stability over multiple years. In return for a flat fee, profit share, or revenue share, they can outsource some or all of their publishing.

#### Partner

The systemic complexity of scholarly communications is mind‐bending, so the transition to OA may be an opportunity to embrace simplicity. Society publishers might consider partnering with one of the following:

- CHORUS (http://www.chorusaccess.org/) and the Jisc Publications Router (https://pubrouter.jisc.ac.uk/) – alternatives to populating individual institutional repositories with accepted manuscripts
- CLOCKSS (https://clockss.org/) and Portico (http://www.portico.org/) – librarians and publishers collaborate to ensure the long‐term digital preservation of journals and other resources crucial to researchers
- COUNTER (http://www.projectcounter.org/about/), DOIs (http://www.doi.org/), ORCID (https://orcid.org/), and more – standardized approaches to reporting usage and identifying content or people help everyone in our ecosystem
- OpenCitations (http://opencitations.net/) – open bibliographic and citation data

#### Society publisher views on cost reduction

**Figure 6:**
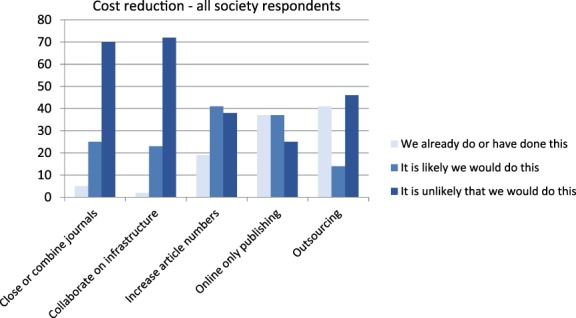
Responses to the question about which cost reduction methods are already in place, and which publishers are likely or unlikely to try (*n* = 104).

**Figure 7:**
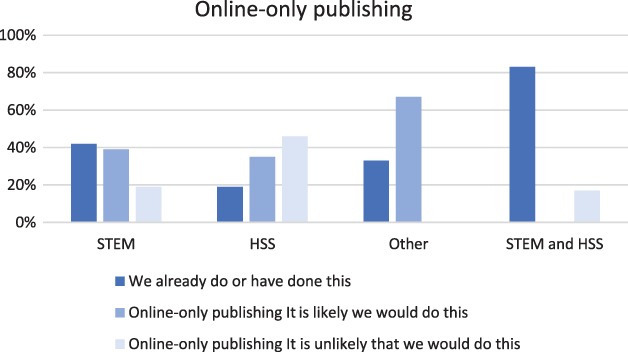
Responses to the question about online publishing only as a cost reduction method; already in place and likely or unlikely reduction (*n* = 99).

Our respondents were generally mindful of costs and had thoughtful responses to questions about whether or not they would use the different approaches and why (see Fig. 6). There did not seem to be much variation between independent publishers and those with larger publishing partners. Differences between geographies were extremely modest, with some suggestion that respondents outside the UK and USA might be more positive about reducing costs by moving online only and less positive about outsourcing. In both cases, there is a relatively low sample size of 15. There were some differences between STEM and HSS publishers in one area, which is their willingness to consider online‐only publishing as a potential way forward (see Fig. 7).

## WHAT WE LEARNED AT OUR FIRST PROJECT WORKSHOP: WHY CAN HYBRID TITLES NOT JUST BE RECOGNIZED AS COMPLIANT WITH PLAN S?

It might be helpful to highlight here the outcome of an unplanned but in‐depth discussion at the first project workshop about why funders will not just agree that hybrid journals should be compliant with Plan S.

Society publishers believed strongly that the hybrid model should be retained as a compliant option for Plan S, along with some form of agreement or control to avoid potential for, or perception of, double‐dipping. In their view, this approach would enable a transition to OA at whatever speed the market naturally evolved.

Funder participants explained that hybrid journals had not led to a full transition to OA. They also pointed out that a very high proportion of access problems with articles that are meant to be OA occur in hybrid titles because OA publishing is shoehorned into systems not designed with support for OA in mind and that librarians remain unconvinced they are receiving value for money and are not paying twice for content that would appear in titles in any case.

Publisher participants described the challenge of flipping hybrid titles with large proportions of unfunded researchers. To aid discussion and understanding, publishers were asked to brainstorm a list of how they defined unfunded researchers. We then worked together to refine this and developed a typology as presented in Table 2. This exercise again reiterated the strong potential strategic value of transformative OA agreements to publishers who seek to transition to full, immediate OA. Publishers were also understandably clear that they cannot be expected to bear all costs for these categories of researchers through APC waivers as this could involve a large proportion of authors for any one title.

**Table 2:** A typology of unfunded researchers.

	Examples
Researchers who are unlikely to have money for APCs but who might potentially be covered by a transformative agreement	University employees: in a team with research funding but with too low a status in the team to access APC funds on teaching contracts or multiple short‐term contracts with grants but no ring‐fenced money for APCs within the grant at institutions that do not support payment of APCs as a valid approach to OA (e.g. due to concern about the potential costs to research‐intensive institutions)
	Researchers affiliated with a university but on an honorary contract
Researchers who are unlikely to have money for APCs and who are unlikely to be covered by a transformative agreement	Researchers employed in organizations that do not primarily engage in research (e.g. colleges, government departments, hospitals, schools, small or specialist universities)
	Retired researchers
	Students
	Unemployed researchers (e.g. recent PhDs)

## RECOMMENDATIONS

In recognition that accelerating OA transitions and aligning with Plan S require change from all stakeholders, we pulled together recommendations from our investigations. Some are for society publishers and others are for funders, larger publishing partners, libraries, and library consortia seeking to help them transition successfully to full and immediate OA.

### Learned society publishers

- Publishers can learn very effectively with and from one another, as well as from other stakeholders. HSS and STEM publishers have much in common. The emergence of groups such as the Society Publishers Coalition is exciting, and trade associations such as ALPSP and The Open Access Scholarly Publishers Association are active in supporting publishers to succeed at OA publishing. Our recommendation is to continue to learn together, collaborate, and pool cost and risk wherever possible.
- In addition to thinking about how to transition to OA, there is real pressure to innovate and drive down costs. Publishers are asked to become expert gymnasts, executing this triple twist with style and panache. There is enough money in the system now for a full transition to OA, but universities are under increasing financial pressure and, in some countries, anticipate funding cuts. At the same time, article numbers are increasing. New services and service providers are constantly emerging with new opportunities to consider. Our recommendation is to embrace this pressure as opportunity and be strategic.
- The pressure for OA from funders, libraries, and some researchers will only grow. As some learned society publishers are demonstrating, the best defence is a good offence. Those publishers that are setting the pace and that are agile and fast will be best placed to try, learn, refine, and succeed. Our recommendation is that all publishers expend effort on action to experiment and find ways to transition to OA.
- Throughout our research, we have seen funders and librarians respond positively and supportively to real concerns by publishers genuinely seeking to transition to OA. We have also seen different responses to some publishers who mainly express defensiveness, frustration, and concern. Our recommendation is that publishers see funders and librarians as potential allies and supporters, as well as customers, and engage proactively and positively.
- Large publishers who publish on behalf of learned society publishers are also grappling with new challenges in the transition to OA. They seek to allocate income fairly while taking into account the geography and subject focus of journals. Although these large publishers were not the focus of the SPA OPS project, recommendations for them arose in the course of our work:
  - There is a real desire by learned society publishers for more information about OA publishing and Plan S requirements. What are the key elements of transformative agreements? What experiments have happened? What worked, what failed, and why?
  - There is potential for new forms of divergence of interest during the transition to OA; learned society publishers desire reassurance that these are being recognized and managed.
  - In particular, they desire transparency around, and more influence over, how revenues from transformative agreements are allocated across proprietary and society‐owned titles.
- While we have found that Transformative Agreements are the most promising mechanism for transition to OA in the short term, publishers should not discard the other approaches and business models outlined in this report and those that are compliant with Plan S requirements. Also very useful are APC models if authors are funded and willing to pay such charges; immediate sharing of accepted manuscripts or final articles under a CC‐BY licence (perhaps deployed only for authors funded by cOAlition S in the first instance and then rolled out more broadly if subscription revenue remains stable); and cooperation, cost savings, and revenue diversification. Very innovative and promising, but perhaps not yet well enough known in the publishing community, are open publishing platforms. Our recommendation is for publishers to consider and experiment with all of these approaches, which they can use alone or in combination.
- It is essential for society publishers to engage authors to supply information about funding sources and institutional affiliations for at least the submitting corresponding authors and to correctly capture and share these metadata.
- Finally, a shift to OA is not only a shift in business models but an opportunity to embrace open principles and values. Increasing transparency and communicating more openly are crucial and may require substantial cultural change. Our recommendation is for publishers to begin increasing transparency about all facets of their publishing with editors, members, and customers.

### Stakeholders seeking to support learned society publishers (e.g. funders, libraries, and library consortia)

- There is wide difference and variation within the publishing industry, and the concerns of independent society publishers may be different from those of society publishers who partner with larger publishers. Our recommendation is to keep talking often and consulting widely and to be seen to do so.
- Journals cannot flip to OA unless their funding streams do. Particular pinch points are a) researcher authors in countries where there is neither funding for APCs nor work underway yet to enter into transformative agreements and b) researcher authors who are at an early stage in their career; clinicians, teachers, and other practitioners; or government employees. Publishers cannot be expected to bear all costs for these categories of researchers through APC waivers, particularly where this could involve a large proportion of authors for any one title.
- Universities provide funding and staff time to publishers and receive contributions and services – particularly from learned societies – in the form of studentships, staff development, teaching, and the like. Our recommendation is that these be tracked in a systematic and more transparent way in order to assess the strategic value of these relationships.
- There is a need for both the public and the private sectors to support OA publishing going forward. For example, commercial organizations that employ authors should fund publication costs for their researcher employees but should also recognize that their readers benefit from the availability of more OA content. Our recommendation is that universities reach out to commercial organizations partnering with their institutions about a shared approach to funding OA publishing. It would also be helpful to engage government departments that fund research and employ researchers but that are not currently actively engaged.
- If all funders worldwide were to align their OA policies, publishers would be able to transition more easily to full and immediate OA. If a majority of funders aligned, then the minority of unfunded articles might be publisher‐funded through waivers. Plan S offers the clearest blueprint for how this can be achieved and is flexible enough to incorporate approaches in the north and south, east, and west. Our recommendation is for funders to work together to support the expansion of aligned OA policies such as Plan S.
- A clearer picture of how scholarly communications might evolve over the medium term could be helpful to all stakeholders. It is easier to look for opportunities to accelerate a transition to OA, and to create a sound foundation for Open Science more broadly, if stakeholders share an understanding of what is likely to happen. Our recommendation is that, before the end of 2020, stakeholders should work together and with publishers on a Political, Economic, Sociological, Technological, Legal, and Environmental (PESTLE) analysis to underpin joint scenario planning about how the transition to OA will proceed over the next 5 years.
- Plan S and initiatives such as OA2020 are powerful because they are drivers for a speedy transition to OA, and indeed for the more fundamental reworking of power relationships and pricing advocated by libraries and universities. Some libraries and consortia have not yet reflected this new urgency in practice, and this could lead some publishers to suspect that the goal is to make them fail. We do not believe this is the case and yet understand the concern. Our recommendation is that work is done to set clear, achievable goals and priorities for the short, medium, and long term and to prioritize pragmatic work to transition to OA while working in parallel on the tougher underlying issues that currently divide stakeholders.
- National differences in subscription pricing have arisen in a historic context – they are based not only on print spend but also on a country's historic ability to pay. Going forward, new approaches and business models need to be transparent and equitable around the world and should be linked to the impact of services on authors, readers, institutions, and society. Rebalancing is likely to be required internationally between countries, and funders are the stakeholder group best placed to enable this. Our recommendation is that societies engage, together and with publishers, in broader strategic discussions about pricing and other factors that could be put in place to ensure a more equitable, innovative, and sustainable scholarly communication system for all.
- The relationship between libraries and society publishers has not previously been a close one. There is now an opportunity for new strategic collaborations as learned societies seek to transition to OA and align with Plan S. Our research shows that transformative agreements are a promising mechanism for this. As library consortia provide the lion's share of funding for the largest players in the current publishing landscape, this could be a very powerful lever for many to accelerate a full transition to OA.
- Finally, this project developed from constructive conversation between funders and publishers. The project steering committee members were extremely collaborative and discussed even challenging subjects in a calm, constructive, and consider way. Our recommendation is that thought be given to how funders, society publishers, and university presses can continue to work through these recommendations and shared challenges during the transition to OA.

## CONCLUSIONS

What should society publishers do to thrive as they transition to OA in alignment with Plan S? Well, quite a lot… it will be a busy few years, but this challenge can be achieved, and there is a huge amount of goodwill and support available.

Perhaps the most important recommendation to society publishers is that they should not think this challenge is going away or that it does not apply to all journal portfolios. We recommend that they:

- Consider all the business models this project has surfaced and do not equate OA with APCs as this will shut down too many options. Of the 27 business models and strategies that our project identified, only 3 rely on author payments to fund article publishing. Transformative agreements, including models such as Subscribe to Open, emerged as the most promising approaches.
- Use the transformative agreement toolkit (Wise & Estelle, 2019e) developed as part of this project to get started. There is no substitute for active, agile learning in order to quickly refine strategy.
- Communicate challenges and opportunities and seek support to overcome or realize them. It is essential that learned society publishers carry their board, editors, and members with them on this journey. Society publishers have willing allies and champions who are ready to help support a successful transition to OA. More transparent communication will help them understand how they can be most supportive and can help address broader issues of trust between research publishers more generally and the broader research community.

## DATA ACCESSIBILITY STATEMENT

Materials and data from the Society Publishers Accelerating Open access and Plan S (SPA OPS) project are available via the Wellcome Trust share platform at https://wellcome.figshare.com/collections/Society_Publishers_Accelerating_Open_access_and_Plan_S_SPA-OPS_project/4561397.
